# Bilirubin and carboxy-hemoglobin concentrations in critically ill patients: prognostic significance of free heme metabolites

**DOI:** 10.1186/cc9785

**Published:** 2011-03-11

**Authors:** H Morimatsu, F Takatsu, J Matsumi, M Tani, Y Moriya, J Kosaka, K Morita

**Affiliations:** 1 Okayama University Hospital, Okayama, Japan

## Introduction

Serum bilirubin is routinely measured in the ICU. Physiologically, bilirubin is one of three heme metabolites such as iron and carbon monoxide (CO), but this fact is almost completely ignored in our daily physiological assessments. In this study, we examined the prognostic significance of these two heme metabolites (T-Bil and CO-Hb) in general ICU populations.

## Methods

We retrospectively studied 723 patients with 12,458 blood gas measurements. Finally, we analyzed paired samples of 1,882 blood gas measurements and laboratory results from 491 ICU patients. We specifically assessed the prognostic significance of serum T-Bil and CO-Hb and their combination.

## Results

**Figure 1:**
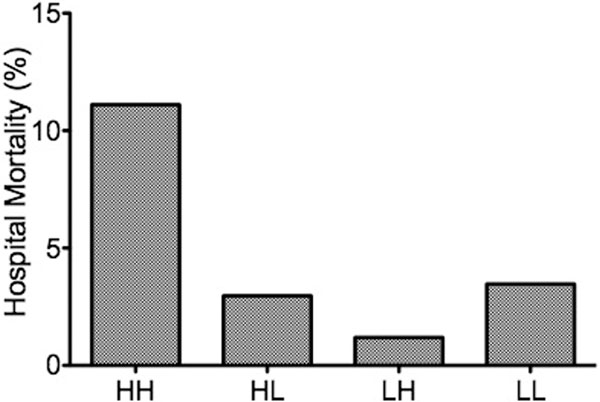
**Hospital mortality divided by T-Bil and CO-Hb levels**.

Our ICU patients had a mean age of 61.8 (SD: 16.1), APACHE II score of 12.1 (4.4). Their hospital mortality was 5.5%. The nonsurvivors had a significantly higher T-Bil compared with the survivors (4.43 (5.30) vs. 1.31 (1.51) mg/dl; *P *= 0.005). On the other hand, a mean of arterial CO-Hb did not differ significantly between the groups (1.52 (0.39)% vs. 1.54 (0.35)%; *P *= 0.86). When patients were divided by four groups according to T-bil (high or low) and CO-Hb (high and low) values, the high-high group had worst outcome (11.1%), but the low-high group had best outcome in the four groups (1.19%) (Figure 1). Finally, prognostic discrimination of T-Bil was significantly improved when arterial CO-Hb was included in the model (area under the ROC curve 0.701 to 0.754).

## Conclusions

Serum T-Bil values were significantly higher in the nonsurvivors than the survivors. Prognostic significance of T-Bil significantly improved when taking into account the CO-Hb levels. These results imply that, even in the general ICU patients, metabolites of heme protein had prognostic significance and importance.
